# Epigenetic Modification and Differentiation Induction of Malignant Glioma Cells by Oligo-Fucoidan

**DOI:** 10.3390/md17090525

**Published:** 2019-09-08

**Authors:** Chien-Huang Liao, I-Chun Lai, Hui-Ching Kuo, Shuang-En Chuang, Hsin-Lun Lee, Jacqueline Whang-Peng, Chih-Jung Yao, Gi-Ming Lai

**Affiliations:** 1 Cancer Center, Wan Fang Hospital, Taipei Medical University, Taipei 11696, Taiwan; 2 Division of Radiation Oncology, Department of Oncology, Taipei Veterans General Hospital, Taipei 11217, Taiwan; 3 National Institute of Cancer Research, National Health Research Institutes, Miaoli 35053, Taiwan; 4 Department of Radiation Oncology, Taipei Medical University Hospital, Taipei Medical University, Taipei 11031, Taiwan; 5 Taipei Cancer Center, Taipei Medical University, Taipei 11031, Taiwan; 6 Division of Hematology and Medical Oncology, Department of Internal Medicine, Wan Fang Hospital, Taipei Medical University, Taipei 11696, Taiwan; 7 Department of Internal Medicine, School of Medicine, College of Medicine, Taipei Medical University, Taipei 11031, Taiwan

**Keywords:** malignant glioma, oligo-fucoidan, differentiation induction, epigenetic modification, DNA methyltransferases

## Abstract

Malignant glioma (MG) is a poor prognostic brain tumor with inevitable recurrence after multimodality treatment. Searching for more effective treatment is urgently needed. Differentiation induction via epigenetic modification has been proposed as a potential anticancer strategy. Natural products are known as fruitful sources of epigenetic modifiers with wide safety margins. We thus explored the effects of oligo-fucoidan (OF) from brown seaweed on this notion in MG cells including Grade III U87MG cells and Grade IV glioblastoma multiforme (GBM)8401 cells and compared to the immortalized astrocyte SVGp12 cells. The results showed that OF markedly suppress the proliferation of MG cells and only slightly affected that of SVGp12 cells. OF inhibited the protein expressions of DNA methyltransferases 1, 3A and 3B (DNMT1, 3A and 3B) accompanied with obvious mRNA induction of differentiation markers (*MBP*, *OLIG2*, *S100β*, *GFAP*, *NeuN* and *MAP2*) both in U87MG and GBM8401 cells. Accordingly, the methylation of *p21*, a DNMT3B target gene, was decreased by OF. In combination with the clinical DNMT inhibitor decitabine, OF could synergize the growth inhibition and *MBP* induction in U87MG cells. Appropriated clinical trials are warranted to evaluate this potential complementary approach for MG therapy after confirmation of the effects in vivo.

## 1. Introduction

Cancer is widely considered as a developmental disease, caused by the dysregulation of cellular proliferation and differentiation [1,2]. Cancer cells generally belong to incomplete cell differentiation mainly in profound impairment of terminal differentiation. Substantial evidence has revealed that this highly de-differentiated and plastic state reflects acquisition of genetic events that actively promote stemness [3]. Tumors thus originate from cells with stem cell characteristics that have acquired aberrant gene expression patterns, mostly due to mutations and epimutations. These aberrant gene expression patterns lead to a block in differentiation and trigger uncontrolled proliferation. Loss of differentiation is thus an important characteristic of tumor cells and represents a defining feature of human cancers. As a consequence, differentiation therapy has been developed into an important approach for the treatment of cancers, particularly hematologic malignancy [4,5].

Malignant glioma (MG) is the most common primary adult brain tumor. According to the 2007 World Health Organization (WHO) classification, gliomas are graded according to the extent of anaplasia (“de-differentiation”) status, with less aggressive designated as WHO grade II, more aggressive forms designated as WHO grade III and the most aggressive one as glioblastoma multiforme (GBM, WHO grade IV). The prognosis of MG remains poor despite a great deal of advances in surgery, radiation and chemotherapy, with a median overall survival of 12–15 months [6,7,8]. The cell origin of MG remains a matter of argument, with evidence indicating it originates from neural stem cells (NSCs), oligodendrocyte precursors (OPCs), or de-differentiated neurons and astrocytes [9,10,11]. Therefore, MG is a developmental disease with incomplete differentiation. Recent studies have demonstrated that substances such as BMPs (bone morphogenetic proteins), Znf179 (a RING (Really Interesting New Gene) finger protein) and CG500354 (a small molecule targeting for cAMP-specific 3′,5′-cyclic phosphodiesterase 4D) are able to reprogram malignant GBM cells to a more-differentiated, less-oncogenic phenotype, which could extend the probability of manipulating the GBM cells toward less-aggressive circumstances [12,13,14]. In addition to hematologic malignancy, MG may also be effectively treated by differentiation therapy. Searching for appropriate effective differentiation inducers is of great importance for this approach. 

Drugs that modulate epigenetic processes in human cancer cells represent an important aspect in the development of differentiation therapy for cancer. All-trans-retinoic acid (ATRA), a well-known differentiation-inducing compound, was among the first substances used for differentiation therapy of acute promyelocytic leukemia [4]. The influential finding that the differentiation-inducing cytosine analogue 2′-deoxy-5-azacytidine (decitabine) acts as an effective inhibitor of DNA methyltransferases provided an important link between cellular differentiation and epigenetic regulation [15,16]. Decitabine is widely used in myelodysplastic syndrome [17]. It also has been evaluated in clinical trials for the treatments of acute myeloid leukemia in recent years [18]. However, many challenges remain in using these epigenetic drugs for the differentiation therapy in solid tumors such as MG. The development of alternative appropriate agents to effectively induce differentiation of MG is needed.

Fucoidan is a natural sulfated polysaccharide found in the cell wall matrix of brown seaweed. Structurally, fucoidan is a heparin-like molecule with a substantial percentage of L-fucose, sulfated ester groups, as well as small proportions of D-xylose, D-galactose, D-mannose and glucuronic acid [19]. Various biological activities of fucoidan, such as antioxidant, anti-inflammatory, antiproliferative and proapoptotic activities have been reported [20,21]. It induces apoptosis in human lymphoma cells by activation of caspase-3 [22], in A549 (human lung adenocarcinoma) cells by activation of caspase-9 [23] and in MCF-7 (human breast cancer) cells by activation of caspases-8 [24], respectively. In addition, fucoidan inhibits invasion and angiogenesis in human fibrosarcoma cells via repression of the activities of matrix metalloproteinases 2 and 9 [25]. Of note, several studies have shown the ability of fucoidan to induce osteoblast differentiation in human osteoblast [26] and adipose-derived stem cells [27]. Furthermore, fucoidan was also reported to stimulate osteoblast differentiation via c-Jun N-terminal kinase (JNK)- and extracellular signal-related kinase (ERK)-dependent bone morphogenetic protein 2 (BMP2)-Smad 1/5/8 signaling in human mesenchymal stem cells [28]. These studies suggest the potential of fucoidan in the differentiation induction of tumor cells, especially MG cells. 

The oligo-fucoidan (OF) used in this study is a low-molecular-weight (<667 Da) fucoidan, which was derived from the glycolytic cleavage product of original fucoidan from brown seaweed *Laminaria japonica* [29]. Various anticancer effects of OF have been reported over the last decade. For examples, the effects of OF against breast and lung cancers via ubiquitin proteasome pathway (UPP)-mediated transforming growth factor β receptor (TGFR) degradation have been demonstrated in animal models by Hsu et al. [30,31]. Our previous study showed that OF regulates miR-29b-DNMT3B-MTSS1 axis and inhibits epithelial–mesenchymal transition (EMT) and invasion in hepatocellular carcinoma cells [32]. In the present study, we explored the effects of OF on the differentiation induction in MG cells and studied the underlying molecular mechanism in the aspect of epigenetic modification. In addition, its combination effects with decitabine, a clinically available demethylating epigenetic agent, in MG cells were also investigated. 

## 2. Results

### 2.1. Oligo-Fucoidan Inhibits Proliferation and Clonogenicity, and Arrests Cell Cycle in Human Malignant Glioma Cells

**Figure 1:**
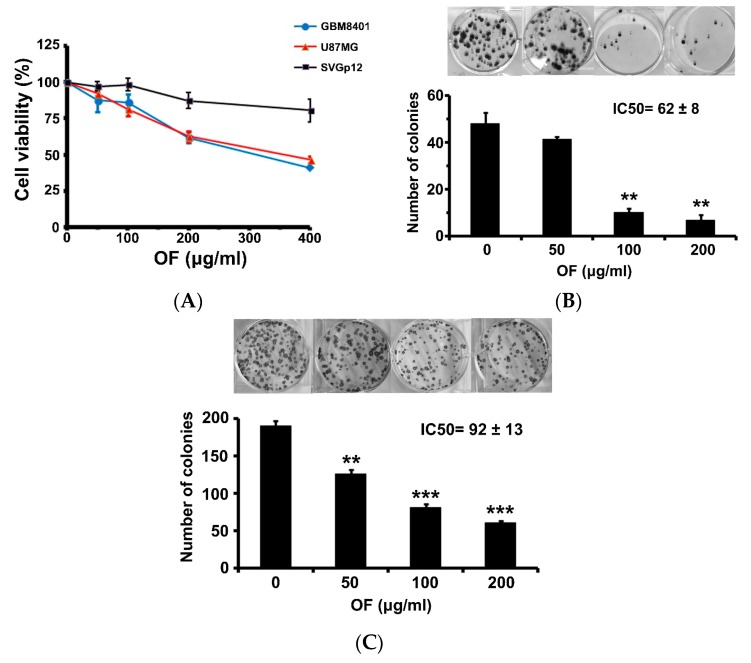
Inhibitory effects of oligo-fucodian (OF) on cell viability and colony formation of human malignant glioma cells. (**A**) Two malignant glioma (MG) cell lines (GBM8401 and U87MG) and immortalized astrocyte SVGp12 cells were treated with various concentrations of OF for 72 h. The cell proliferation was measured by sulforhodamine (SRB) assay. Values are expressed as the mean ± standard error of triplicate wells. (**B**) Effects of OF on the clonogenicity of GBM8401, and (**C**) U87MG cells. Each experiment was performed in triplicate, and the representative examples are shown (column, mean, bar, standard error; ** *p* < 0.01; *** *p* < 0.001). The IC_50_ indicates the 50% inhibitory concentration (μg/mL) of OF in the 12-day clonogenicity assay of GBM8401 and U87MG cells, respectively. Data are expressed as mean ± standard error.

The effect of OF on the proliferation of human MG cells (GBM8401 and U87MG) determined by sulforhodamine (SRB) assay is shown in Figure 1. Varying degrees of growth inhibition were observed after 72 h exposure to OF. At a concentration of 400 μg/mL, the cell growth of GBM8401 and U87MG cells were inhibited to 40% and 46% of the control, respectively (Figure 1A). In contrast, OF only had a slight inhibitory effect on the growth of immortalized astrocyte SVGp12 cells at the same concentration, suggesting the preferential suppression of cancer cells by OF. At concentration of 200 μg/mL, OF significantly decreased the colony formation of GBM8401 and U87MG cells to 14% and 32%, respectively (Figure 1B,C). The 50% inhibitory concentration (IC_50_) of OF in clonogenicity of GBM8401 and U87MG cells upon 12-day treatment was 62 ± 8 and 92 ± 13 μg/mL, respectively (Figure 1B,C). A higher grade of MG cells seemed to be more sensitive to OF.

**Figure 2:**
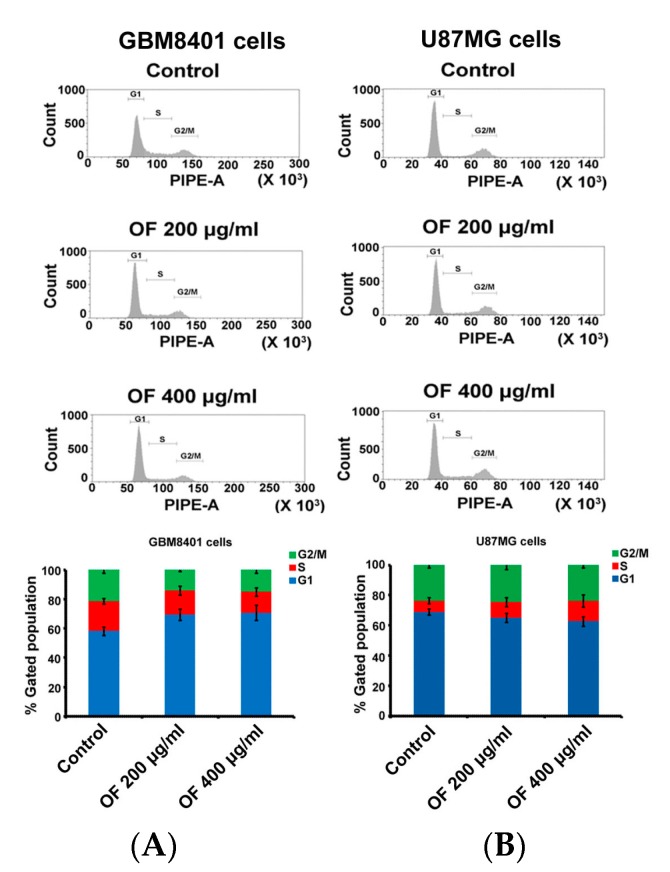
Analysis of cell-cycle distribution in malignant glioma cells after treatment with oligo-fucoidan (OF). After 72 h treatment, the effects of OF on cell-cycle distributions of GBM8401 (**A**) and U87MG (**B**) cells were analyzed by flow cytometry. The quantitative measurement of G1, S and G2/M phases of GBM8401 and U87MG cells after treating with OF.

Figure 2A,B show the cell-cycle distribution of GBM8401 and U87MG cells after treatment with OF at concentrations of 200 and 400 μg/mL for 72 h. OF arrested the cell cycle of GBM8401 cells by increasing the proportion of G1 phase from 58% (control) to 69% and 71%, respectively (Figure 2A). In U87MG cells, OF concentration dependently increased the S phase from 7% (control) to 10% and 14%, respectively (Figure 2B). The results indicate that in different types of MG cells, OF could inhibit proliferation via arresting the cell cycle at either the G1 or S phase. 

### 2.2. Oligo-Fucoidan Induces Differentiation of Malignant Glioma Cells

**Figure 3:**
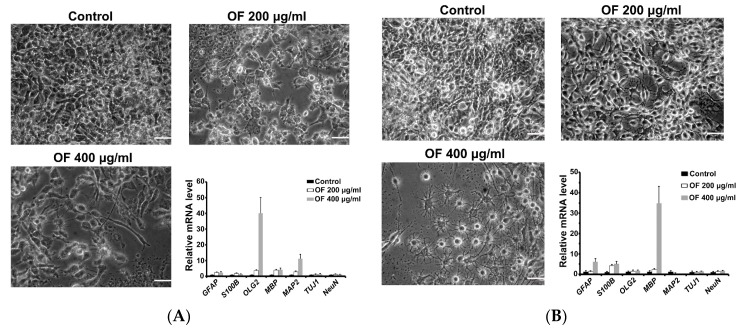
Differentiation induction of human MG cells after treatment with oligo-fucodian (OF). (**A**) GBM8401 and (**B**) U87MG cells were treated with OF for seven days. Morphology of the MG cells was examined by inverted phase contrast microscopy. Scale bar is 50 μm. Expression of differentiation marker genes was analyzed by quantitative PCR. Astrocyte markers: *GFAP* and *S100B*; oligodentrocyte markers: *Olig2* and *MBP*; neuron markers: *MAP2*, *TUJ1* and *NeuN*. Data were expressed as mean ± standard error.

As shown in Figure 2, apoptosis induction was not observed in OF-treated MG cells. Nonetheless, marked changes of cellular shape to the morphologies of neural, oligodendrocyte or glial cells were displayed after treatment with OF. This suggests that OF-mediated inhibition of MG cells might attribute to differentiation induction rather than cytotoxic effect. To confirm this assumption, a panel of early (astrocyte (GFAP), oligodendrocyte (Olig2) and neuron (MAP2 and Tuj1)) and terminal (astrocyte (S-100β), oligodendrocytes (myelin basic protein, MBP) and neuron (NeuN)) differentiation markers were measured in OF-treated MG cells by quantitative PCR assay. As shown in Figure 3A, neural and oligodendrocyte-like cellular shapes were observed in OF-treated GBM8401 cells. In support of this, early differentiation markers of oligodendrocytes (*Olig2*) and neurons *(MAP2*) were markedly elevated in these GBM8401 cells (Figure 3A). In OF (400 μg/mL)-treated U87MG cells, more oligodendrocyte-like and less glial-like cellular shapes were observed (Figure 3B). In accordance, a dramatic elevation of terminal oligodendrocyte differentiation marker *MBP* and significant increase of astrocyte markers (*GFAP* and *S100B*) were detected in these U87MG cells (Figure 3B). Together, OF might induce re-differentiation of MG cells, which were driven to malignant transformation by the de-differentiation events described in Section 1.

### 2.3. Oligo-Fucoidan Inhibits DNA Methyltransferases (DNMTs) in Human Malignant Glioma Cells

**Figure 4:**
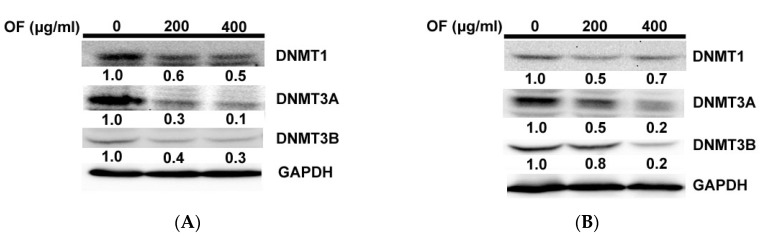
Oligo-fucodian (OF) represses protein levels of DNA methyltransferases (DNMTs) in human MG cells. After treatment with OF for 72 h, the protein levels of DNMT1, DNMT3A and 3B in GBM8401 (**A**) and U87MG (**B**) cells were analyzed by Western blot analysis. Glyceraldehyde-3-phosphate dehydrogenase (GAPDH) was used as the loading control for Western blot analysis. The fold changes of these protein levels were indicated below the band.

Next, we investigated the molecular mechanism underlying the differentiation of OF-treated MG cells. Epigenetic modulation involving DNA demethylation was known to play a crucial role in the differentiation of MG cells [33]. Our previous study found that OF is able to induce miR-29b [32], which suppresses DNMTs (DNMT1, 3A and 3B) in cancer cells [34]. We thus determined if the DNMTs of MG cells were inhibited during differentiation induction by OF. As expected, OF repressed the protein levels of DNMTs in both GBM8401 (Figure 4A) and U87MG (Figure 4B) cells. Epigenetic mechanism involving DNA demethylation might play a crucial role in the differentiation induction by OF.

### 2.4. Oligo-Fucoidan Decreases the Methylation of p21 Gene Accompanied with Induction of Its Expression in Human Malignant Glioma Cells

**Figure 5:**
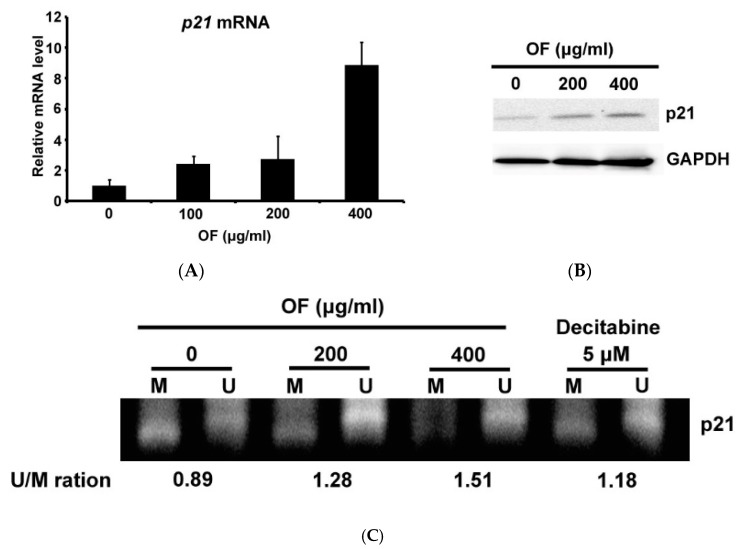
Oligo-fucodian (OF) increases p21 expression and decreases *p21* gene methylation in human MG cells. The *p21* mRNA (**A**) and p21 protein (**B**) levels of U87MG cells were increased after treatment with OF for seven days. The mRNA and protein levels were analyzed by quantitative PCR and Western blot analysis, respectively. (**C**) Analysis of *p21* gene methylation by methylation-specific PCR. PCR products amplified with methylated (M) and unmethylated (U) sequence-specific primers were shown. The quantitative U/M ratios are indicated below each pair of bands. Decitabine was used as positive control.

The expression of *p21* (*CDKN1A*, cyclin dependent kinase inhibitor 1A) is known to be repressed by DNMT3B through methylating the CpG islands in its promoter region [35]. Regarding the substantial inhibition of DNMT3B protein level by OF in U87MG cells, it may decrease the methylation of *p21* gene and restore its expression. In agreement, OF induced the mRNA (Figure 5A) and protein (Figure 5B) levels of p21 in U87MG cells in a concentration-dependent manner. Through further examination of the methylation status of *p21* gene by methyl-specific PCR, we found that OF increased the proportion of unmethylated (U) p21 promoter and decreased the methylated (M) (Figure 5C). The U/M ratio in control U84MG cells was 0.89 and increased to 1.28 and 1.51 by OF at concentrations of 200 and 400 μg/mL, respectively (Figure 5C). The known demethylating agent decitabine (5-aza-2′-deoxycytidine) was used as a positive control, which increased the U/M ratio of *p21* gene from 0.89 to 1.18 at concentration of 5 μM (Figure 5C). As *p21* is a tumor suppressor and cyclin-dependent kinases (CDK) inhibitor, epigenetic induction of its expression would play a crucial role in OF-mediated growth inhibition in MG cells. It is considered that the repressed differentiation marker genes might be epigenetically induced by OF through the similar mechanism of action in demethylation. 

### 2.5. Combination with Oligo-Fucoidan (OF) Enhances Decitabine-Mediated Growth Inhibition and Differentiation Induction in MG Cells

**Figure 6:**
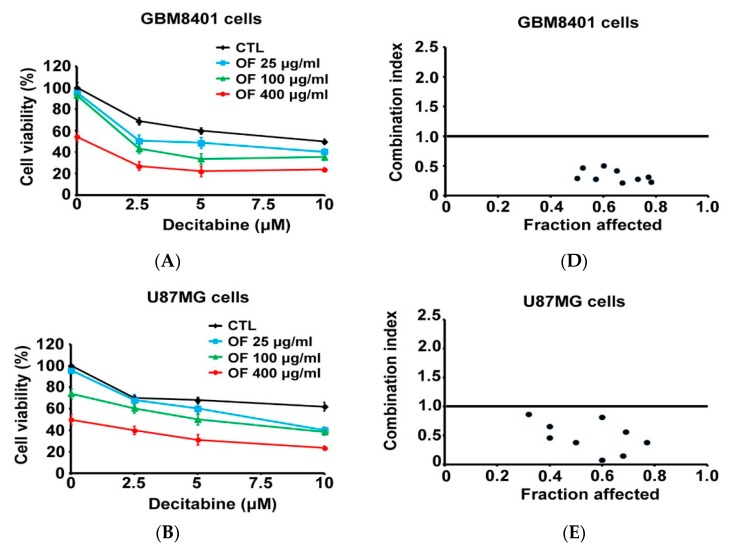

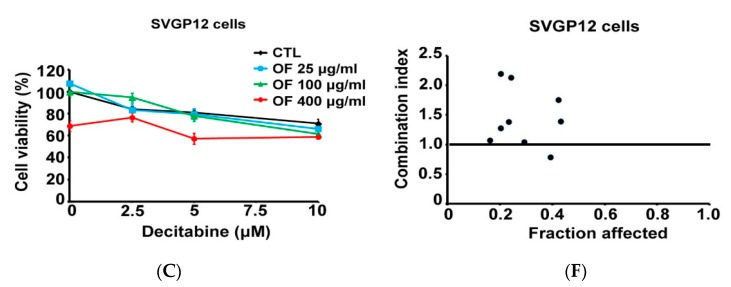
Combination effects of oligo-fucoidan (OF) and decitabine on the proliferation of MG cells. (**A**–**C**) GBM8401, U87MG and immortalized human astroglia (SVGp12) cells were treated with various concentrations of decitabine in combination of OF as indicated for 72 h. The proliferation of cells was measured by sulforhodamine (SRB) assay. Values are expressed as the mean ± standard error of triplicate wells. (**D**–**F**) Combination index values of decitabine–OF combinations vs. the inhibition (fraction affected) of cell proliferation. Values lower than 1 show synergistic effects, whereas those equal or close to 1 are additive and those higher than 1 are antagonistic.

Aberrant methylation of DNA has been proposed as a target for novel cancer treatment [36]. However, substantial hurdles (low efficacy and high toxicity) limit the extension of this proposal to solid tumors [36]. Considering the substantial demethylating effect of OF shown in Figure 5C, its combination with decitabine might be an alternative way for the epigenetic therapy of MG via demethylation. As shown in Figure 6A,B, decitabine only slightly inhibited the growth of GBM8401 and U87MG cells to 64% and 70% of the control, respectively, even at the maximum concentration of 10 μM. When combined with OF (400 μg/mL), decitabine decreased the growth of GBM8401 and U87MG cells to less than 40% of the control (Figure 6A,B). In contrast, the immortalized astrocyte SVGp12 cells were much less sensitive to the combination effect of decitabine and OF (Figure 6C), suggesting the selectivity of this combination against MG cells. The combination effect was further examined by the so-called combination index (CI), which quantitatively depicts synergism (CI < 1), additive effect (CI = 1) and antagonism (CI > 1) [37]. As shown in Figure 6D,E, the CI values in both GBM8401 and U87MG cells were all below 1, indicating synergisms of this combination against the proliferation of MG cells. By contrast, the CI values of decitabine combined with OF in SVGp12 cells were above 1 (Figure 6F), suggesting an antagonism effect on cell proliferation. 

**Figure 7:**
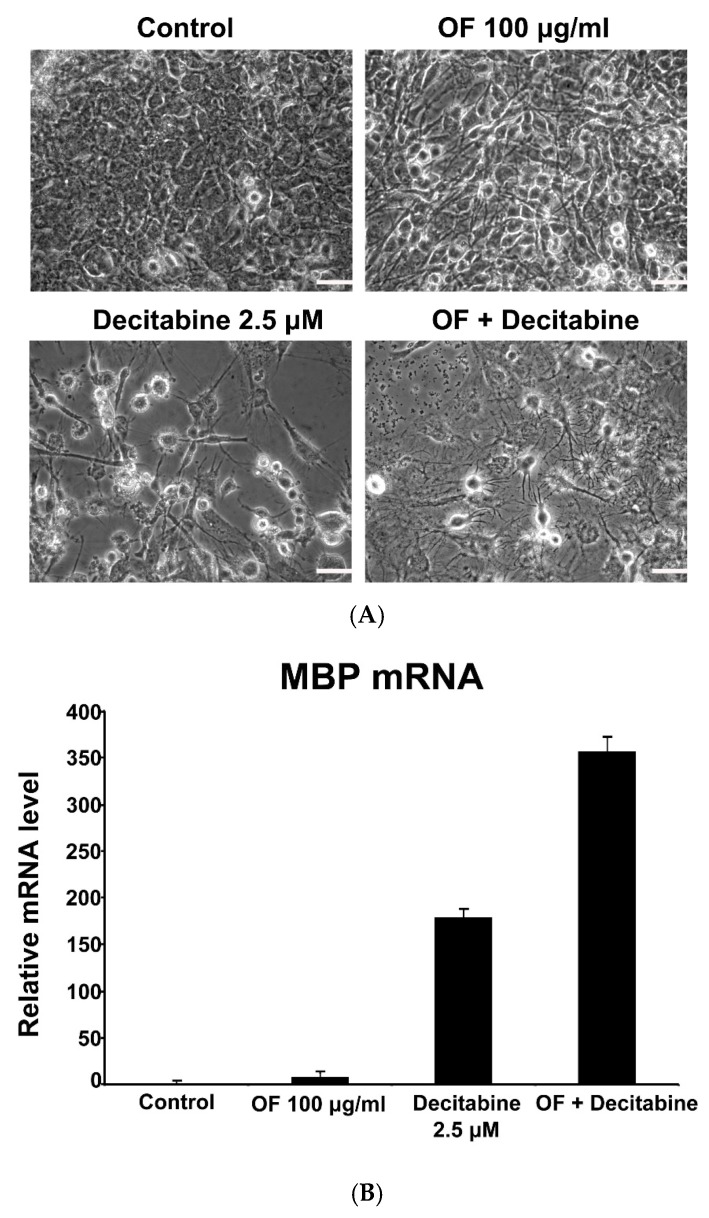
Combination effects of oligo-fucoidan (OF) and decitabine on the differentiation induction of MG cells. (**A**) U87MG cells were treated with OF (100 μg/mL), decitabine (2.5 μM) or a combination of each other for seven days. Morphology of the MG cells was examined by inverted phase contrast microscopy. Scale bar is 50 μm. (**B**) U87MG cells were treated with OF (100 μg/mL), decitabine (2.5 μM) or a combination of each other as indicated for seven days. The gene induction of differentiation marker *MBP* was analyzed by quantitative PCR.

In line with the combination effect on growth inhibition, the differentiation induction of U87MG cells by decitabine at 2.5 μM was also markedly enhanced by OF at 100 μg/mL. As shown in Figure 7A, the display of oligodendrocyte-like morphology induced by OF or decitabine in U87MG cells was markedly enhanced in the combination-treated group. In support of this, OF or decitabine alone induced the mRNA expression of terminal differentiation marker *MBP* to 7 and 180 folds of the control, respectively (Figure 7B). In combination-treated U87MG cells, the expression of *MBP* was markedly enhanced to 357 folds of the control (Figure 7B). Thus, consistent with that observed in morphologic changes, combining OF and decitabine has an obvious synergistic effect on not only the inhibition of cell proliferation, but also the differentiation induction in MG cells. 

## 3. Discussion 

Fucoidan is a botanical sulfated polysaccharide extracted from brown seaweed. Its widespread bioactivity has gained significant research attention. Previous studies have reported that the in vitro anticancer activity of low-molecular-weight fucoidan (<5000 Da) was significantly higher than that of native fucoidan (>30 kDa) from sporophyll of *Undaria pinnatifida* [38,39]. However, the sulfate contents in both fucoidans are similar [38], suggesting that the anticancer activity of fucoidan could be considerably improved by lowering the molecular weight. As such, we chose the low-molecular weight (<667 Da) oligo-fucoidan (OF) [29] as the research material in this study. 

Numerous researches have reported various anticancer effects of OF in many different types of tumor cells, including hepatoma, breast cancer and lung adenocarcinoma cells [30,31,32]. However, as far as we know, no literature has shown the effects of OF on MG cells. In this study, we demonstrated the growth arrest of MG cells by OF, accompanied with induction of differentiation marker genes expression. Current treatments of MG remain focused on achieving maximal surgical resection followed by concurrent radiation therapy with temozolomide. Conventional treatment offers patients with GBM additional survival time with generally acceptable quality of life, but a cure is never achieved [40]. In addition to killing cancer cells by conventional chemotherapy or radiotherapy, reactivation of their endogenous differentiation program to resume the maturation process and abolish tumor phenotypes has been proposed [41]. However, its application in MG treatment is hampered by the toxicity and limited efficacy of current clinically-used differentiation agents such as ATRA, azacitidine and decitabine in solid tumors [42,43]. Hopefully, our results in this study might help to shed light on the approach of MG differentiation therapy.

In response to previous reports showing the effects of fucoidan to stimulate osteogenic differentiation of adipose-derived stem cells [27] and mesenchymal stem cells [28], we demonstrated the effects of OF on differentiation induction in MG cells, accompanied with simultaneous DNMTs inhibition. It has been hypothesized that in glioma hypermethylator phenotype tumors, the extensive DNA methylation maintains MG cells in a de-differentiated state [33]. In support of this, differentiation induction and growth inhibition in IDH1 (isocitrate dehydrogenase 1) mutant MG cells by the DNMT inhibitor decitabine has been demonstrated [33]. Therefore, the down-regulation of DNMTs in OF-treated MG cells suggests the epigenetic mechanisms involving DNA demethylation for this OF-mediated differentiation induction. Moreover, it has been reported that DNMT1 and DNMT3B are overexpressed in gliomas and inhibiting DNMTs by 5-azacytidine (azacytidine, DNMT inhibitor) enhances expression of tumor suppressor genes such as p21 [44]. Accordingly, we found the induction of *p21* accompanied the decrease of its methylated promotor region in OF-treated U87 MG cells. Our previous work showed the inhibition of DNMT3B by OF via miR-29b induction [32]. As miR-29b has been shown to directly target DNMT3A and 3B and indirectly down-regulate DNMT1 by targeting Sp1 [34], miR-29b induction might participate in OF-mediated inhibition of DNMTs in MG cells. The epigenetic modification activities of OF make it an attractive option for the complementary management of MG.

There is evidence that epigenetic regulation plays a crucial role not only in cell differentiation and embryonic development, but also in the self-renewal of cancer stem cells [45]. Exploring the manipulation of epigenetic networks may provide new insights for differentiation therapy in solid tumors [45]. In contrast to genomic mutations, epigenetic changes remain reversible and have been targeted by agents such as DNA methylation inhibitor decitabine (5-Aza-2’-deoxycytidine) and histone deacetylase inhibitor suberoylanilide hydroxamic acid (SAHA; vorinostat) in cancer clinical trials [46]. In brain cancers, many loci exhibit epigenetic alterations and therapies to reverse these changes are thus being pursued [46]. As there are a lack of effective DNA demethylating agents with low toxicity and ease of delivery to the brain, clinical trials for epigenetic therapies of MG are currently limited to SAHA [46]. To promote clinical trials in brain tumors, combining inhibitors of DNA methylation and histone deacetylation has been proposed to reduce toxicity and increase efficacy through their synergistic effects at lower doses [46]. In line, OF combined with chidamide, a histone deacetylase inhibitor, could synergistically inhibit growth of MG cells (Supplementary Data 1). Moreover, synergistic induction of oligodendrocyte terminal differentiation marker MBP in U87MG cells was achieved by combination of decitabine and OF. Consistently, OF also synergized the effect of decitabine against the proliferation of GBM8401 and U87MG but not the immortalized astrocyte SVGp12 cells. It is expected that epigenetic therapies for MG will be established in the near future [45]. Our results suggest the potential of combining OF and currently-used epigenetic drugs as the complementary approach for this goal. 

As mentioned above, epigenetic regulation also plays a crucial role in the self-renewal of cancer stem cells [45], which are more resistant to conventional chemotherapy and radiotherapy than more differentiated tumor cells [47]. In agreement of this, we found that OF could reduce the elevated stem markers in the cancer stem-like U87 sphere cells, resulting in the diminishment of sphere number and size, and the reduction of cancer stem-like side population percentage (Supplementary Data 2). Triggering the differentiation of cancer stem cells has been proposed to restore their sensitivities to regular chemotherapy and radiotherapy [47]. Regarding the epigenetic- and differentiation-inducing effects of OF shown in this study, it might be able to enhance the efficacy of chemotherapy and radiotherapy for MG and warrants further investigation. In parallel, previous studies have shown the enhancing effects of fucoidan on the activities of chemotherapeutic agents, such as tamoxifen, cisplatin and paclitaxel against cancer cells [48,49]. In glioblastoma, it has been proposed that targeting the cancer stem cells rather than the bulk tumor mass would be more effective [47]. Combination with OF might be an alternative way to enhance the chemotherapeutic effects against MG stem cells and is worthy of further investigation. 

On the other hand, whether OF can pass the blood-brain barrier (BBB) is critical for its application in differentiation therapy of MG. The BBB is constituted from cerebrovascular endothelial cells through forming complex tight junctions, which obstruct the passing of chemical agents to enter the brain. The tissue distribution of naïve fucoidan after intragastric administration to rats had been studied by Pozharitskaya et al. [50]. In their study, the average molecular mass of the naïve fucoidan was estimated to be 735 kDa [50], which is much higher than the upper limit (400 Da) [51] for efficient permeability through the BBB. Pozharitskaya et al. did not analyze the brain distribution of the fucoidan they used [50]. As aforementioned, the OF used in the present study is a low-molecular-weight (<667 Da) fucoidan, which was derived from the glycolytic cleavage product of original fucoidan from brown seaweed *Laminaria japonica* [29]. The molecular weight of OF is much smaller than that of the naïve fucoidan (735 kDa) [50] and is near the upper limit (400 Da) [51] for efficient permeability through the BBB. Moreover, in brain tumors, the increased permeability of the BBB by disrupting endothelial tight junction proteins via vascular endothelical growth factor (VEGF) in GBM [52] and the fenestration and vesicles in the capillary endothelium of pilocytic astrocytomas [53] have been found. These abnormalities in the BBB of MG suggest the possibility for OF to pass through. Ultimately, an appropriate animal study is warranted to further confirm the OF-mediated differentiation of MG in vivo. 

**Scheme 1:**
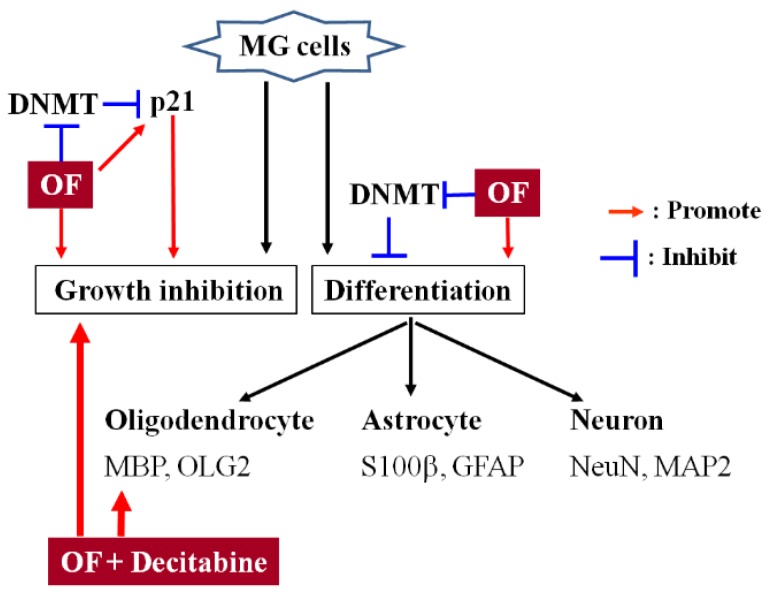
Schematic diagram displays the proposed mechanisms of action of OF (oligo-fucoidan) in MG (malignant glioma) cells.

In summary, our results demonstrate the induction of OF-mediated DNMTs inhibition and differentiation markers in MG cells (Scheme 1). Its synergistic combination effects with decitabine against MG cells suggest a potential complementary approach for the epigenetic differentiation therapy of MG. After confirmation of the effects in vivo, an appropriated clinical trial is warranted to evaluate its clinical benefit for MG.

## 4. Materials and Methods 

### 4.1. Reagents

The fucoidan powder from *Laminaria japonica*, a commercial product named Hi-Q Oligo-fucoidan^®^, was provided by Hi-Q Marine Biotech International Ltd. (New Taipei City, Taiwan). Briefly, the crude extract of *Laminaria japonica* was eluted with a NaCl gradient on a DEAE (Diethylaminoethyl)-Sephadex A-25 column. The fucose- and sulfate-enriched fraction eluted at a higher concentration of NaCl was collected and then hydrolyzed with glycolytic enzyme preparation to obtain our oligo-fucoidan (OF) sample [54]. The characteristics of oligo-fucoidan (OF) were as follows: average molecular weight of <667 Da with a 85.9% fucose content (127.2 ± 1.3 μmol/g), sulfate content 28.4% ± 2.1% (*w*/*w*), protein content 4.3% ± 0.3% (*w*/*w*), fat content 0.6% ± 0.1% (*w*/*w*), ash 4.1% ± 0.1% (*w*/*w*) and moisture content 3.9% ± 0.8% (*w*/*w*) [29]. It was dissolved in double-distilled H_2_O and stirred at 25 °C for 30 min. The dissolved solution was filtered using 0.22 μm sterile filters (Millipore, Billerica, MA, USA). Decitabine (5-aza-2′-deoxycytidine, Cat #A3656, Sigma-Aldrich, St Louis, MO, USA) was used as a demethylating agent. Stock solutions of decitabine (20 mM) were dissolved in dimethyl sulfoxide (Cat #D2650, Sigma-Aldrich).

### 4.2. Cell Culture

The human glioblastoma multiforme cell line GBM8401 (BCRC 60163) was purchased from the BCRC (Bioresource Collection and Research Center, Hsin Chu, Taiwan). Human glioblastoma cell line U87MG was obtained from American Type Culture Collection (Manassas, VA, USA). GBM8401 cells were maintained as monolayers in Dulbecco’s modification Eagle’s medium (DMEM, Gibco, CA, USA) and U87MG cells were maintained in Roswell Park Memorial Institute (RPMI) 1640 (Gibco). These culture mediums supplemented with 10% fetal bovine serum (Gibco) and 1× penicillin-streptomycin-glutamine (PSG, Gibco). Cells were cultured at 37 °C in a water jacketed 5% CO_2_ incubator.

### 4.3. Cellular Viability

Cells were seeded in 96-well plates at a density of 2000 cells per well and treated with various concentrations of oligo-fucoidan (OF) for 3 days. At harvest, the cell numbers were determined by sulforhodamine (SRB) that measured the cellular protein content. Briefly, cells were fixed in 10% trichloroacetic acid and stained with 0.4% SRB (Sigma-Aldrich). After incubation and washing, bound SRB was dissolved in 100 μL of 10 mM unbuffered Tris base and optical density was measured at 570 nm using a microtiter plate reader (ELx800; BioTek, Winooski, VT, USA). At least three independent measurements were performed for each experiment.

### 4.4. Clonogenicity

Cells were plated at a density of 200 cells/well in 6-well plate. After seeding for 24 h, cells were treated with indicated agents for 12 days. At the end, cells were stained with crystal violet (Sigma-Aldrich), photographed and the colonies that expanded to >50 cells were counted.

### 4.5. Photograph of the Cells

The phase contrast images of cells were photographed using a digital microscope camera (PAXcam2+, Villa Park, IL, USA) adapted to an inverted microscope (CKX31; Olympus, Tokyo, Japan) at 40× objective lens magnification. 

### 4.6. Cell-Cycle Distribution Analysis

Cells were seeded in 6-cm dishes at a density of 4 × 10^5^ per dish. After treatment with OF at indicated concentrations for 72 h, cells were trypsinized, washed twice by PBS, fixed in ice-cold 70% ethanol and stored at 4 °C. The cells were then washed twice with ice-cold phosphate-buffered saline and then incubated with RNase and DNA intercalating dye propidium iodide (50 μg/mL) at room temperature for 20 min. The cell-cycle distributions were then analyzed using a CytoFLEX flow cytometer (Beckman Coulter). A minimum of 10,000 events were collected and analyzed.

### 4.7. Quantitative RT-PCR

Total RNA was extracted from untreated or oligo-fucoidan (OF)-treated malignant glioma cells, using an RNeasy Mini Kit, and treated with an RNase-free DNase I set (Qiagen, Hilden, Germany) according to the manufacturer’s protocol. Total RNA (1 μg) was reverse-transcribed using oligo (dT)_15_ primers and a reverse transcription system (Promega, Madison, WI, USA). Reactions were carried out using Fast SYBR^®^ Green PCR Master Mix (Applied Biosystems, Warrington, UK) on the Step One Plus Real-Time PCR System (Applied Biosystems, Foster City, CA, USA) by denaturation at 95 °C for 10 min, followed by 40 cycles at 95 °C for 15 s and 60 °C for 40 s. Melting curve analyses were performed to verify the amplification specificity. Relative quantification of gene expression was performed according to the ΔΔ-CT (threshold cycle) method using StepOne Software 2.0 (Applied Biosystems). The sequences of qPCR primers used to probe differentiation marker genes of astrocyte, oligodendrocytes and neurons are listed in Table S1. Glyceraldehyde-3-phosphate dehydrogenase (GAPDH) was used as an internal control.

### 4.8. Western Blotting

Cell extracts were prepared from cells that were suspended in Radio-Immunoprecipitation Assay (RIPA) lysis buffer with protease inhibitor (Roche, Pleasanton, CA, USA). After centrifugation, supernatants were dissolved in the Laemmli sample buffer (Bio-Rad, Hercules, CA, USA) for sodium dodecyl sulfate-polyacrylamide gel electrophoresis (SDS-PAGE). Approximately 50 μg of protein were separated in SDS-PAGE and electrotransferred onto a Polyvinylidene Fluoride (PVDF) membrane. The membrane was blocked with 5% skim milk and then probed with the following primary antibodies: anti-DNMT1 (ab13537, Abcam MA, USA), anti-DNMT3A (GTX129125, Gene Tex, Irvine, CA, USA), anti-DNMT3B (GTX129127, Gene Tex), anti-GAPDH (ab8245, Abcam) and anti-p21 (ab109199, Abcam) at 4 °C for overnight. After incubation with horseradish peroxidase-conjugated secondary antibody (Jackson Immunoresearch, West Grove, PA, USA), the membrane was then visualized using Immobilon Western Chemiluminescent HRP Substrate (Millipore, Burlington, MA, USA). The Western blotting results were quantified with Image J software (NIH, Bethesda, MD, USA).

### 4.9. DNA Isolation and Sodium Bisulfite Conversion

Genomic DNA was isolated from malignant glioma cells after treatment with oligo-fucoidan (OF) using the QIAquick kit (Qiagen, Germantown, MD, USA). Bisulfite conversion of genomic DNA was performed using EZ DNAMethylation-Gold^TM^ kit (Zymo Research, Irvine, CA, USA) following the manufacturer’s instruction. After bisulfite treatment, unmethylated cytosines were converted to uracil (which was amplified as thymidine in subsequent PCR assays), whereas methylated cytosine remained unaltered.

### 4.10. Methylation Specific PCR (MSP)

The methylation status of *p21* genes was assessed by using conventional methylation specific PCR. Appropriate primer pairs (*p21* methylation forward primer, 5′-TACGCGAGGTTTCGGGATC-3′; reverse primer, 5′-CCCTAATATACAACCGCCCCG-3′; *p21* unmethylation forward primer, 5′-GGATTGGTTGGTTTGTTGGAATTT-3′; reverse primer, 5′-ACAACCCTAATATACAACCA CCCCA-3′) were employed. The presence of methylated cytosine residues was indicated by an amplification product using the primer pair specific for methylated DNA. The reaction mixture was preheated at 95 °C for 5 min, followed by 40 cycles at 95 °C for 30 s, 60 °C for 45 s, 72 °C for 30 s and the final step at 72 °C for 5 min.

### 4.11. Synergistic Combination Effect

The synergism between OF and decitabine on the growth inhibition of cancer cells was analyzed by the combination index (CI) derived from the median effect principle of Chou and Talalay [55], using the CalcuSyn software (version 1.1.1; Biosoft, Cambridge, UK). The value of CI < 1 points to a synergism effect, whereas the value of CI = 1 points to an additive effect and CI > 1 indicates an antagonism effect.

### 4.12. Statistical Analysis

Differences between the clonogenicity data of control and treated groups were evaluated by one-way ANOVA followed by Dunnett’s *t-*test. Probability value of *p* < 0.05 was considered statistically significant. Single asterisk (*) indicates *p* < 0.05; double asterisks (**) indicate *p* < 0.01; triple asterisks (***) indicate *p* < 0.001.

## Supplementary Materials

The following are available online at https://www.mdpi.com/1660-3397/17/9/525/s1, Figure S1: Combination effects of Oligo-Fucoidan (OF) and Chidamide on the proliferation of MG cells, Figure S2: Effects of OF on the cancer stemness of U87MG cells, Table S1: Primers used in Real-Time PCR analyses, Experimental Procedures: Sphere formation assay and Side Population Analysis.
