# Supplementary material for: Epigenetic Modification and Differentiation Induction of Malignant Glioma Cells by Oligo-Fucoidan
Source: Mar Drugs. 2019 Sep 8;17(9):525. doi: 10.3390/md17090525 (PMC6780514; doi:10.3390/md17090525)
Supplement: Supplementary file 1 [file marinedrugs-17-00525-s001.pdf]

Supplementary information

1. Experimental Procedures

Sphere formation assay

The spheres were collected by gentle centrifugation, then dissociated with trypsin-EDTA and mechanically disrupted with a pipette. The resulting single cells were then centrifuged to remove the enzyme and re-suspended in serum-free medium allowed to re-form spheres. The cells were subsequently cultured in ultra-low attachment 6-well plates at a density of no more than 5000 cells/well. After culture for 10 days, spheres were quantitated by inverted phase contrast microscopy.

Side Population Analysis

The U87MG cells were detached from the dishes with Trypsin-EDTA (Invitrogen) and suspended at 1×10^6 cells/mL in Hank's balanced salt solution (HBSS) supplemented with 3% fetal calf serum and 10 mM HEPES. These cells were then incubated at 37°C for 90 minutes with 5 μM Vybrant® DyeCycle™ Violet (Invitrogen, Grand Island, NY, USA), either alone or in the presence of 2.5 μM reserpine (Sigma-Aldrich, MO, USA), which is an inhibitor of ABC transporter. After 90-minute incubation, the cells were centrifuged immediately for 5 minutes at 300×g, 4°C and resuspended in ice-cold HBSS. The cells were kept on the ice to inhibit efflux of DCV and 1 μg/mL propidium iodide (BD Pharmingen, San Diego, CA) was then added to discriminate dead cells. Finally, these cells were filtered through a 40 μm cell strainer (BD Falcon) to obtain single-suspension cells. Cell dual-wavelength analysis was performed on a Cytoflex flow cytometer (Beckman Coulter).

2. Supplemental figure and figure legends

(A) (B)

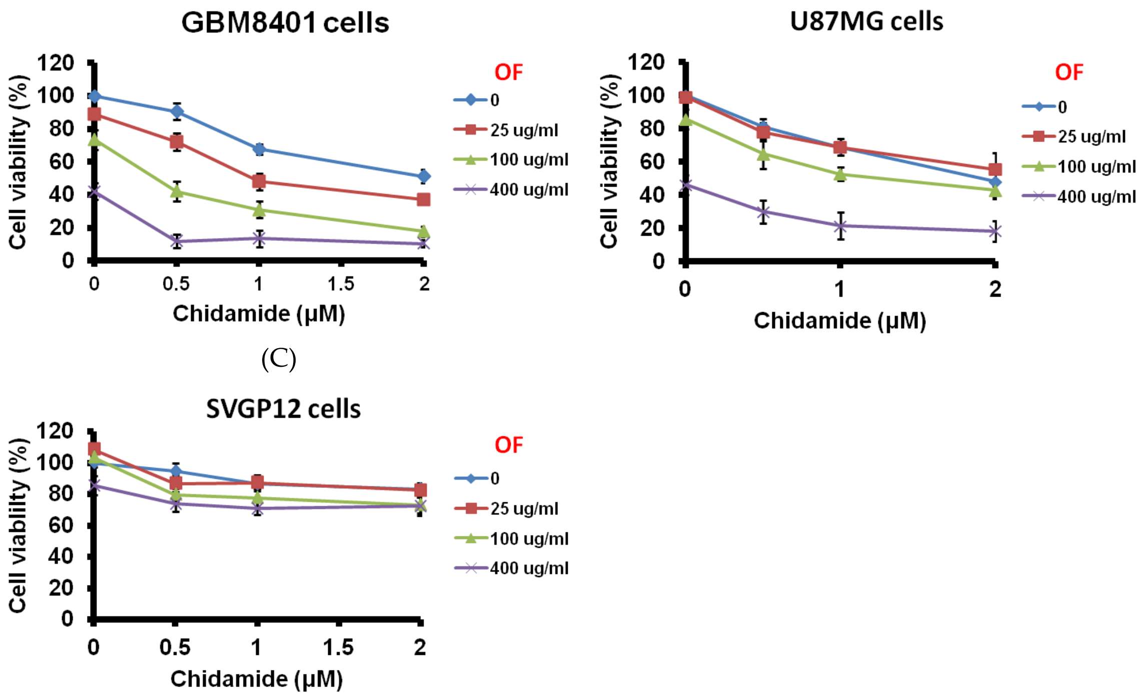

Figure S1. Combination effects of Oligo-Fucoidan (OF) and Chidamide on the proliferation of MG cells. (A–C) GBM8401, U87MG and immortalized human astroglia (SVGp12) cells were treated with various concentrations of Chidamide in combination of OF as indicated for 72 h. The proliferation of cells was measured by sulforhodemine (SRB) assay. Values are expressed as the mean ± standard error of triplicate wells.

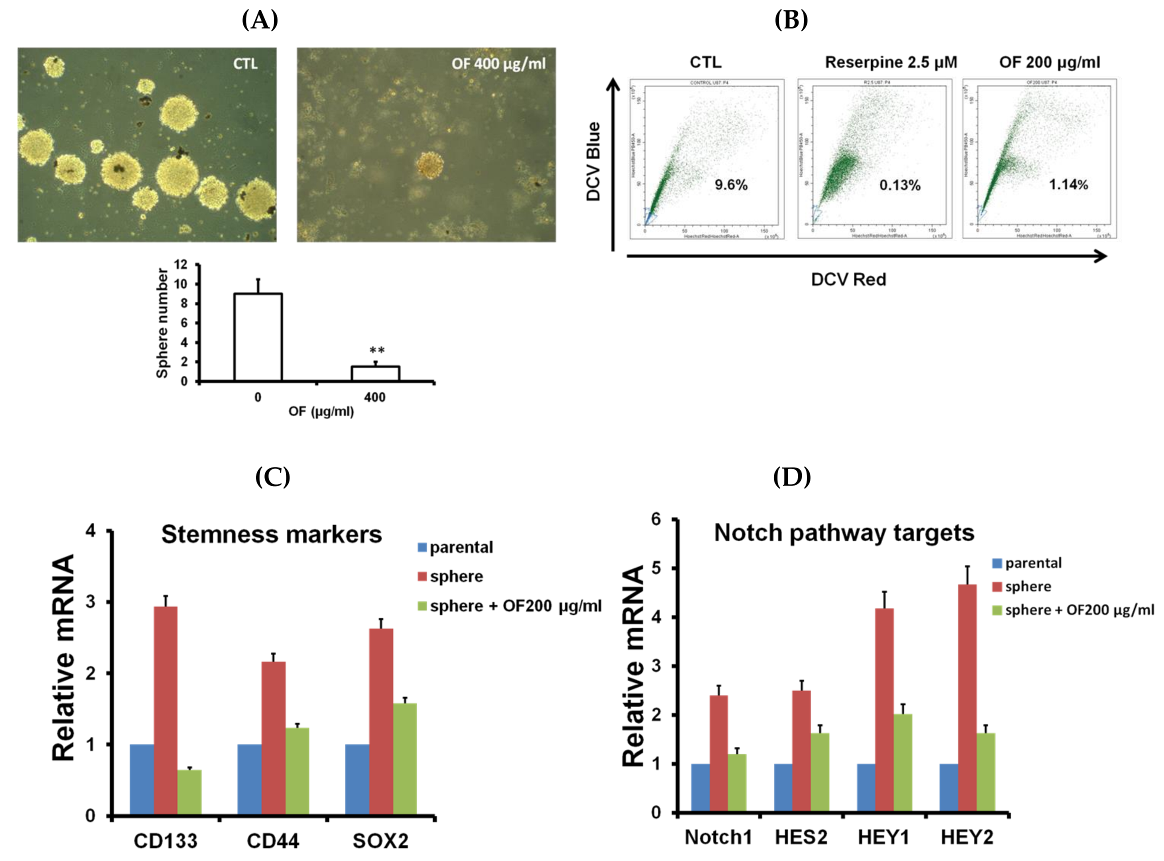

Figure S2. Effects of OF on the cancer stemness of U87MG cells.
(A) U87MG sphere-forming cells with stem cell-like properties were treated with 400 μg/ml OF for 7 days. Phase contrast image of spherical colonies treated with OF versus untreated control are shown. The sphere formation numbers of the OF-treated groups were significantly inhibited in comparison to controls. (B) U87MG cells were incubated with 200 μg/ml of OF for 3 days, and then the proportion of cancer stem-like side population (SP) was gated and analyzed after staining with DyeCycle™ Violet (DCV). The SP cells which disappeared in the presence of reserpine (intermediate panel) are outlined and shown as a percentage of the total cell population. (C) U87MG sphere-forming cells were treated with 200 μg/ml of OF for 5 day, and then the mRNA levels of stem cell markers and Notch pathway targets (D) were analyzed by Q-PCR.

Table S1: Primers used in Real-Time PCR analyses.

Gene	Primer (Forward)	Primer(Reverse)
GAPDH	GTGGACCTGACCTGCCGTCT	GGAGGAGTGGGTGTCGCTGT
GFAP	ACATCGAGATCGCCACCTAC	ACATCACATCCTTGTGCTCC
S100B	TGGCCCTCATCGACGTTTTC	ATGTTCAAAGAACTCGTGGCA
OLIG2	CCAGAGCCCGATGACCTTTTT	CACTGCCTCCTAGCTTGTCC
MBP	GGCCGGACCCAAGATGAAAA	CCCCAGCTAAATCTGCTCAGG
MAP2	CTGCTTTACAGGGTAGCACAA	TTGAGTATGGCAAACGGTCTG
TUJ1	GGCCAAGGGTCACTACACG	GCAGTCGCAGTTTTCACACTC
NEUN	CCAAGCGGCTACACGTCTC	CGTCCCATTCAGCTTCTCCC
p21	TGTCCGTCAGAACCCATGC	AAAGTCGAAGTTCCATCGCTC
CD133	GCATTGGCATCTTCTATGGTT	CGCCTTGTCCTTGGTAGTGT
CD44	CAGCAACCCTACTGATGATGACG	GCCAAGAGGGATGCCAAGATGA
SOX2	CACATGAACGGCTGGAGCAA	GGAGTGGGAGGAAGAGGTAAC
Notch1	ATCGGGCACCTGAACGTGGCG	CACGTCTGCCTGGCTCGGCTC
HES2	AACCAGAGCCTGAGCCAGCTTA	TGCAGGAAGCGCACGGTCATTT
HEY1	TGTCTGAGCTGAGAAGGCTGGT	TTCAGGTGATCCACGGTCATCTG
HEY2	TGAGAAGACTTGTGCCAACTGCT	CCCTGTTGCCTGAAGCATCTTC
